# How limb dominance influences limb symmetry in ACL patients: effects on functional performance

**DOI:** 10.1186/s13102-022-00579-y

**Published:** 2022-12-07

**Authors:** F. Zumstein, C. Centner, R. Ritzmann

**Affiliations:** 1 Praxisklinik Rennbahnklinik AG, Kriegackerstrasse 100, 4132 Muttenz, Switzerland; 2 Department of Sport and Sport Science, University of Freiburg, Freiburg, Germany

**Keywords:** ACL, Limb dominance, Limb symmetry index, Return to sport, Functional testing

## Abstract

**Background:**

Timing for return to sport (RTS) after anterior cruciate ligament (ACL) injury is paramount for the avoidance of a secondary injury. A common criterion in RTS decision-making is the limb symmetry index (LSI) which quantifies (a)symmetries between the affected and unaffected limb. Limb dominance is one of many factors that may contribute to the recovery of the LSI after ACL reconstruction. The purpose of this study was to examine how limb dominance affects the LSI of functional performance tasks nine months following ACL reconstruction (time of RTS).

**Methods:**

At time of return to sport, *n* = 100 patients (*n* = 48 injured the dominant limb, *n* = 52 injured the non-dominant limb, *n* = 34 female, *n* = 66 male) with ACL reconstruction surgery performed isokinetic strength measurements of the knee extensors and flexors, and drop jumps (DJ), single leg hop for distance (SHD) and 6 m timed hop (6MTH) testings.

**Results:**

The findings indicated that injury of the dominant leg led to significantly higher LSI values in maximal isokinetic knee extensor strength (*p* = 0.030). No significant differences were observed for maximal isokinetic knee flexor strength, DJ, SHD or 6MTH performance. Stratifying for sex revealed no significant differences. Simple regression analyses demonstrated that LSI in maximal knee extensor strength significantly predicted LSIs in DJ and SHD while explaining 14% and 18% of the respective variance.

**Conclusions:**

Given that limb dominance affects the LSI of muscle strength suggests that a differentiated interpretation of the LSI with respect to limb dominance should be considered for a safe return to sport. Monoarticular knee extensor strength and multiarticular hop test performance are interrelated and thus can show asymmetries which are not maladaptive but established during years of habituation or training.

**Supplementary Information:**

The online version contains supplementary material available at 10.1186/s13102-022-00579-y.

## Introduction

With an annual incidence of 69 per 100′000 individuals, isolated anterior cruciate ligament (ACL) tears are one of the most common orthopedic injury [1]. Women are more affected than men [2]. Still up to 75% get surgically reconstructed [1], what leads to extended rehabilitation times [3]. To reduce the risk of reinjury and to evaluate the adequate time for athletes to safely return to their sport, progressive rehabilitation programs which are time- and criteria based moved into clinical focus [4, 5]. The progress is governed by movement diagnostics which deliver objective results (i.e. knee extensor and flexor strength, coordination, balance, movement quality) to assess the rehabilitation progress.

Previous research has uniformly demonstrated that patients with ACL rupture experience substantial strength impairments of up to 30% following six months after injury and reconstruction compared to the contralateral leg [6]. Additionally, due to its high correlations with the development of osteoarthritis [7], strength assessments are playing an integral role within the holistic functional diagnostics in ACL patients. Beyond assessments of muscle strength, hop tests are frequently conducted to evaluate proper knee function due to their high similarity to sports specific movements and high functionality [8, 9]. Among the variety of hop tests, the single leg hop for distance (SHD), drop jump (DJ) and the 6 m timed hop (6MTH) are most commonly used in patients following ACL reconstruction and demonstrated high reliability scores [10–16]. Within these tests, both movement quality as well as quantitative measures (e.g., jumping distance, time or force vectors) are analysed.

From a return to sport point of view, it is generally postulated that a successful rehabilitation is defined by the regaining of symmetric functioning of the lower extremity [3, 5, 10, 12, 16, 17] at nine month following surgery [3]. The quantification of (a)symmetries are typically expressed via the limb symmetry index (LSI) as the score between the injured and non-injured leg ((LSI = value of the affected leg: value of the unaffected leg) × 100%) [18]. To evaluate the readiness for return to sport, most researchers and practitioners set the cut-off value generally at 90% [10–13, 15]. The cut off is set independently of sex although women demonstrate higher incidences for ACL ruptures [19] but lower ones for re-ruptures [20]. The comparison with the healthy, contralateral limb minimizes probable confounding variables of the biological variation between people [21], and is due to its feasibility widely used in practice [10, 15, 22]. In a study by Noyes et al. [23], the authors showed that a 10% difference (i.e., LSI 90%) in one-legged hop tests is within the normal range for healthy, uninjured persons. Further, healthy students demonstrated significant differences between the dominant and non-dominant limb for the SHD, 6MTH and the triple hop performance [24] suggesting a potential effect of limb dominance during functional tasks.

With regard to functional diagnostics during ACL rehabilitation, injuries at the non-dominant limb inherently increase side differences between both legs, which might result in a reduced LSI. However, if the dominant (and mostly stronger) leg is affected, the loss in muscle strength leads to an alignment in the force levels between the dominant and non-dominant leg. When interpreting LSI, this must be considered since this might erroneously lead to higher symmetry values compared to baseline. The lack of including limb dominance in this interpretation can thus lead to overestimation and therefore to premature return to sport with an elevated risk of reinjury [25].

Therefore, the aim of the study was to examine how limb dominance affects the LSI of various functional performance tasks in patients after ACL reconstruction at the time of return to sport (i.e. nine months after ACL reconstruction). We hypothesized that the LSIs are significantly higher for patients who injured the dominant leg than for patients who injured the non-dominant leg. In a secondary analysis, the effect of sex as well as the interaction between limb dominance and sex was investigated on the LSI measures.

## Methods

### Design

**Fig. 1:**
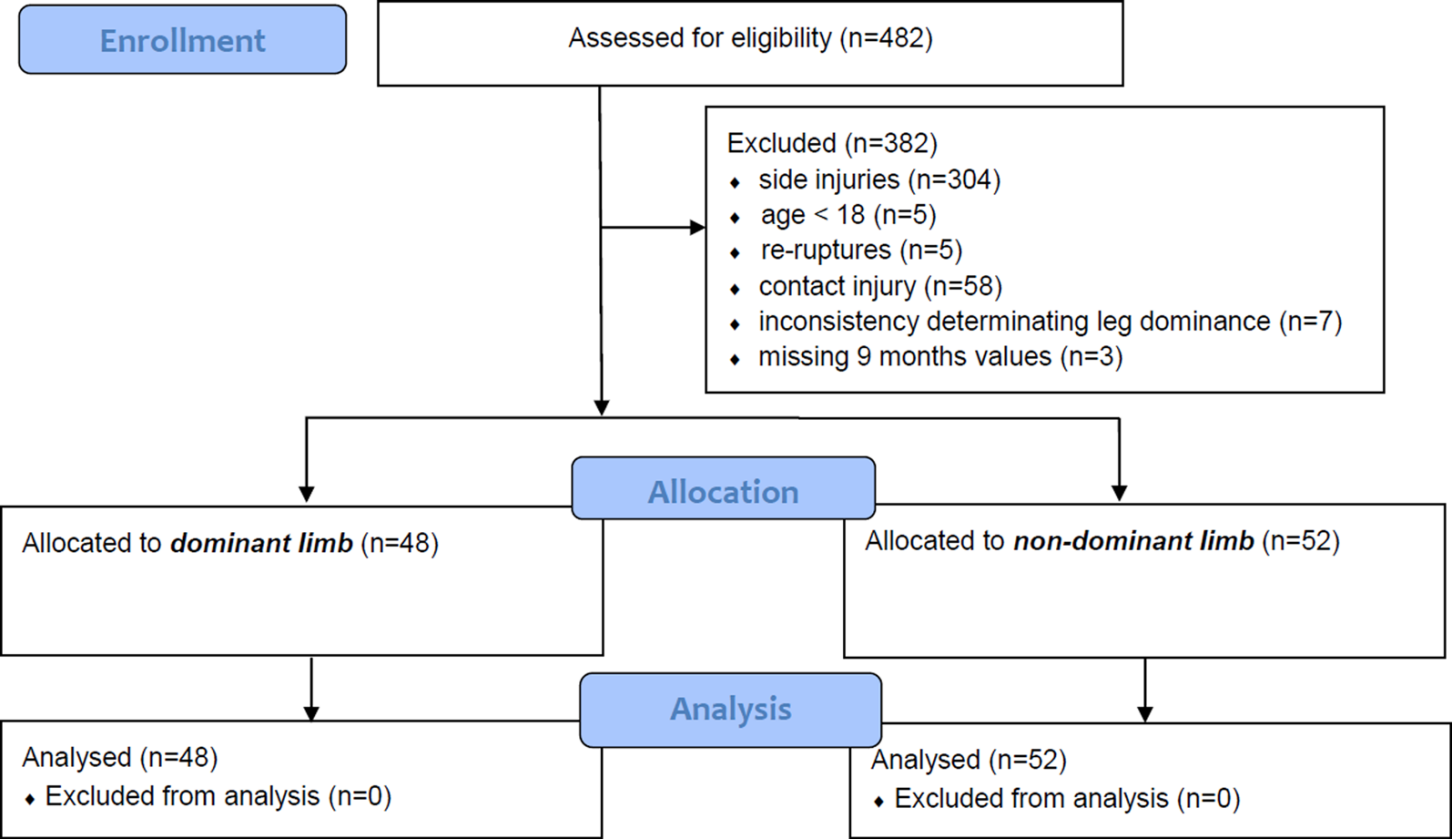
Flow chart

This is a retrospective cross-sectional study based on experimental patient medical records of patients being included for date of surgery from 01.02.2018 to 31.03.2020. The time of data acquisition was nine months post ACL surgery. The dataset contains information from a consecutive sample of patients, stratified by age, sex, and limb dominance undergoing ACL reconstructive surgery at a specialized sports orthopedic hospital. Patients were eligible for inclusion into the study if: (a) aged between 18 and 60 years and b) arthroscopically assisted ACL reconstruction using a quadrupled, single-bundle ipsilateral semitendinosus autologous graft. Exclusion criteria were: (a) age under 18 or over 60, (b) additional knee surgeries which affect rehabilitation time (meniscal suture, axis corrections, reconstructive cartilage interventions [26, 27]), (c) ACL re-ruptures, (d) neurological disorders, (e) inconsistency defining limb dominance three and six month after injury (in case of both dominant sites), (f) missing values nine month after ACL reconstruction surgery, and (g) full contact injuries (Flow chart diagram Fig. 1). Full contact injuries were defined according to Brophy et al. and were excluded as they are considered to impose a mechanical stress in the ligamentous structure that cannot be sustained or avoided [28].

The study was approved by the local ethics committee (2021-01106) and conducted in accordance with the latest revision of the Declaration of Helsinki [29]. All testing data were encoded to ensure anonymity of the participants. The specialty of the hospital entails the spectrum of surgical joint reconstructions for the lower and upper extremities. These include state-of-the-art procedures on ligaments, tendons, bones, cartilage and muscles, as well as therapeutic follow-up nine months after surgery.

### Subjects

From a total of *n* = 482 screened records, *n* = 100 subjects (66 males, 34 females) were enrolled who met all above inclusion criteria and experienced an arthroscopically guided primary ACL reconstruction surgery at the Praxisklinik Rennbahn AG, Muttenz, Switzerland. To increase homogeneity, post-surgical treatment and rehabilitation was also standardized by a time- and criteria evidence-based rehabilitation [3]. Physical rehabilitation was performed two times a week by a physical therapist. A detailed description of the entire rehabilitation protocol can be found elsewhere [30]. Briefly, the rehabilitation protocol included passive and active mobilisation interventions, sensorimotor training, strength training, jumping, cutting and pivoting manoeuvres training for a duration of 9 month [10, 31–33]. The goal of all rehabilitation was a successful return to sport defined as reaching the functional competence of the pre-injury level. Limb dominance was determined by the question with which foot they would kick a ball [34–36].

Participants’ characteristics are presented in Table 1.

**Table 1:** Patient characteristics and anthropometric data

Groups	Dominant Group	Non-dominant Group	*p*-values
Number	n = 48	n = 52	
Male	**n = 34**	**n = 32**	
Female	**n = 14**	**n = 20**	
Right	n = 45	n = 3	
Left	n = 3	n = 49	
Data assessment (in weeks after surgery)	40.68 (± 4.264)	40.2 (± 3.541)	0.537
*Anthropometrics*			
Age (years, m ± SD)	32.33 (± 9.85)	28.61 (± 8.83)	0.050
Body mass (kg, m ± SD)	80.15 (± 15.5)	79.46 (± 15.71)	0.829

Data underlined in bold illustrate the interaction between sex and limb dominance

### Performance measures

The testing sessions were performed at the time of return to sport after 40.4 ± 3.9 weeks. For familiarization prior to each testing session subjects completed an identical and standardized 10 min warm up protocol on a cycling ergometer (~ 50 W). Isokinetic strength measurements, DJ, SHD and timed hop tests were executed in a randomized order, all trials started with the unaffected limb.

#### Isokinetic strength measurements

To assess isokinetic unilateral knee extensor and flexor strength an isokinetic dynamometer (Humac^®^/NormTM Testing & Rehabilitation System, Computer Sports Medicine, Inc.; CSMi, Stoughton, Massachusetts, US) was used [37]. Patients were seated in a rigid chair and strapped at the thorax, hip and distal thigh. The trunk angle kept stable at 85°. The rotational axis of the dynamometer was aligned to the lateral femoral epicondyle, with a fixation of the lower leg to the dynamometer lever arm at the medial malleolus. Three submaximal trials were performed for familiarization, followed by two sets of five repetition with maximal effort [30]. All trials were performed from maximal flexion to maximal extension starting with the unaffected limb. Angular velocity was 60°/s. The average of the two best repetitions was calculated and normalized to body mass (Nm/kg) [30]. As outcome parameters the peak extension and flexion torques were extracted and used for LSI analysis [38].

#### Drop jumps (DJs)

DJs were performed on one leg from a 20 cm high platform on top of a bilateral force plate (MLD, SpSport, Kriens, Austria) [39]. Subjects were instructed to perform drop jumps with stiff legs, a short ground contact time and a rapid push-off with maximal height [40]. Three submaximal trials were performed for familiarization for each side. Subsequently, the two best out of three trials were selected and ground reaction forces were normalized to the body mass (N/kg). The average of these trials for each limb was used to calculate the LSI.

#### Single leg hop for distance (SHD)

The aim of the SHD is to hop forward as far as possible, while maintaining a controlled landing on the ipsilateral limb. Technical considerations have been respected according to literature [10–16]. The distance was measured from the start line to the heel of the landing limb, and the LSI was expressed as the ratio between affected and unaffected limb.

#### Timed hop test

For the timed hop test, the goal was to hop on a single limb as quickly as possible over a distance of 6 m [10–16]. For both hop tests two trials were obtained for each limb. The average of the trials for each limb was used to calculate the LSI.

### Statistical analysis

All statistical analyses were conducted with R [41] and figures were produced using the packages ggplot [42] and sjPlot [43].

Normal distribution was confirmed using Shapiro–wilk test and visual inspection of QQ-plots. Homogeneity of variances was assessed using Levene’s test. Differences between both limb dominance groups (dominant vs. non-dominant) were examined using unpaired t-tests and chi-squared test (for the nominal variable of sex). Furthermore, a general linear model with the factors age, sex, limb dominance and limb dominance*sex was calculated to investigate potential interaction effects between limb dominance and sex on limb symmetry measures.

To investigate to what extent knee extensor LSI predicts jump performance, a simple linear regression was performed for both dominant and non-dominant groups.

All following data are presented as mean ± SD if not otherwise stated. The alpha level was set to *p* < 0.05.

## Results

Out of the n = 100 patients with arthroscopic ACL reconstruction, n = 48 injured the dominant limb and n = 52 the non-dominant limb (Table 1). There were no significant differences between the groups (dominant vs. non-dominant) in any of the anthropometric variables (*p* > 0.05).

After stratifying for sex, there was no significant difference in the distribution with respect to limb dominance; 51.5% of males injured the dominant limb as compared to 41.2% of the females (*p* = 0.327).

**Fig. 2:**
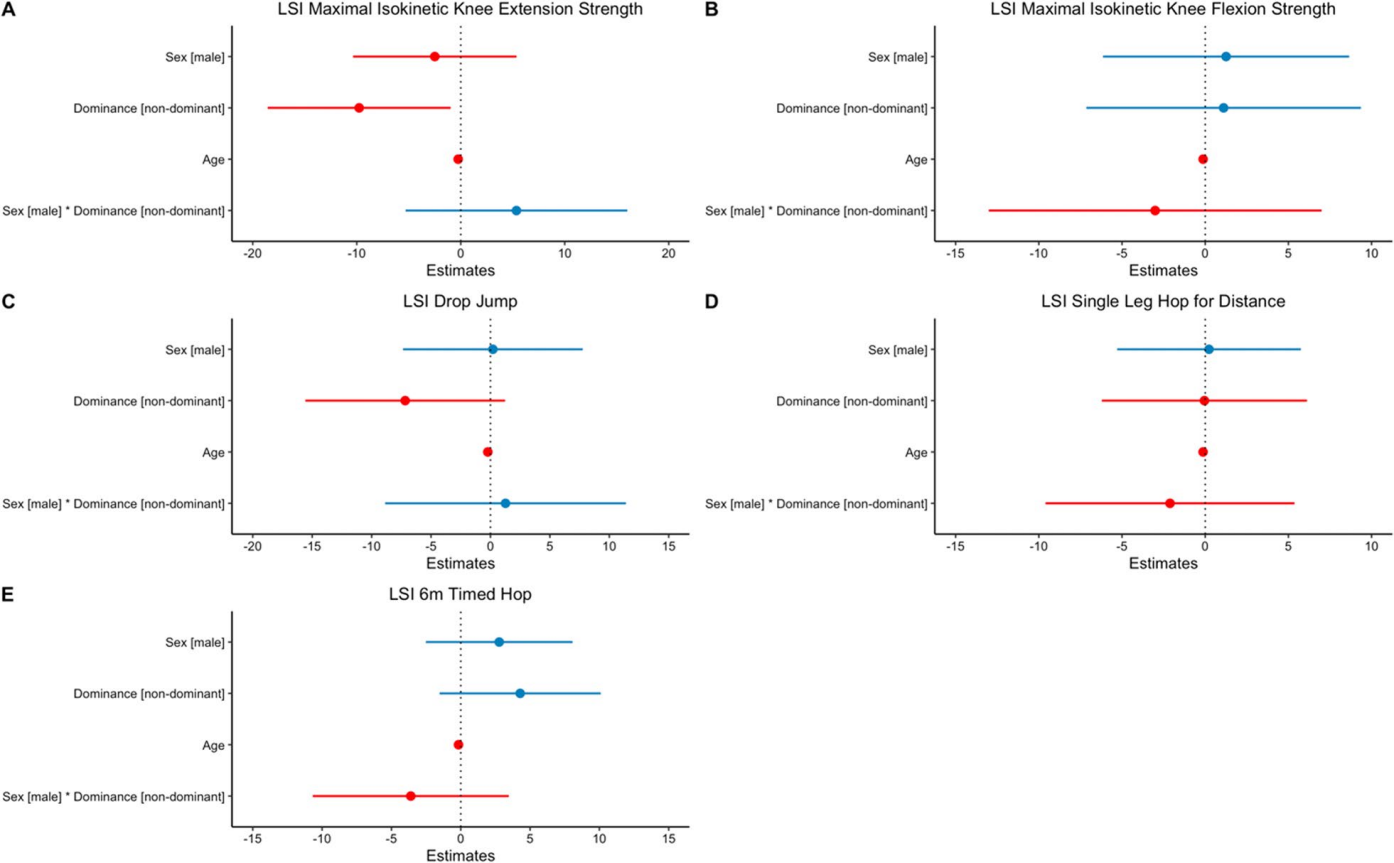
Point estimates and 95% confidence intervals for sex, limb dominance, age and sex*limb dominance interaction on LSI maximal isokinetic knee extension strength (**A**), LSI maximal isokinetic knee flexion strength (**B**), LSI drop jump (**C**), LSI Single Leg Hop for Distance (**D**) and 6 m timed hop (**E**). Colors indicate positive (blue) and negative (red) point estimates

The main effect of limb dominance regarding the LSI of the patients who injured their dominant limb were significantly higher for the maximal isokinetic knee extensor strength (*p *= 0.030) (Fig. 2). There was no significant between-group difference for the maximal isokinetic knee flexor strength (*p* = 0.790), the DJ (*p* = 0.094), SHD (*p* = 0.988) or the 6MTH (*p* = 0.147) (Table 2 and Fig. 2). Stratifying for age and sex as well as sex*limb dominance interaction did not reveal any significant differences (all *p* > 0.05, Fig. 2 and Table 3). Detailed results of regression models are depicted in the supplementary material (Additional file 1).

**Table 2:** Mean differences between the dominant and non-dominant group

Limb Symmetry Index	Dominant	Non-dominant	Statistics (*p*-value)
Knee extensor strength [%]	90.72 (± 12.65)	85.34 (± 12.25)	*p* = 0.030*
Knee flexor strength [%]	90.29 (± 12.37)	89.90 (± 10.89)	*p* = 0.790
Drop Jump [%]	94.52 (± 11.44)	88.96 (± 11.88)	*p* = 0.094
Single Leg Hop for Distance [%]	94.55 (± 7.92)	93.58 (± 9.19)	*p* = 0.988
6 m timed Hop [%]	94.55 (± 7.92)	93.58 (± 9.19)	*p* = 0.147

Limb symmetry indices of maximal isokinetic knee joint extensor and flexor torques [%], DJ [%], SHD [%] and 6MTH [%] are illustrated as means ± standard deviations (m ± SD).

*indicates a significant main effect of limb dominance

**Table 3:** Interaction between limb dominance and sex

Limb Symmetry Index	Dominant Group		Non-dominant Group		Statistics (*p*-value)
	Male	Female	Male	Female	
Knee extensor strength [%]	90.12 (± 13.34)	92.14 (± 11.18)	86.29 (± 13.18)	83.79 (± 10.70)	*p* = 0.321
Knee flexor strength [%]	90.71 (± 11.32)	89.29 (± 15.04)	89.10 (± 11.06)	91.15 (± 10.78)	*p* = 0.552
Drop Jump [%]	94.68 (± 11.28)	94.14 (± 12.32)	89.36 (± 11.99)	88.36 (± 12.00)	*p* = 0.804
Single Leg Hop for Distance [%]	94.72 (± 8.19)	94.16 (± 7.56)	92.77 (± 9.27)	94.89 (± 9.15)	*p* = 0.576
6 m timed Hop [%]	95.8 (± 7.59)	92.75 (± 8.37)	96.92 (± 6.33)	97.96 (± 10.94)	*p* = 0.314

Limb symmetry indices [%] of maximal isokinetic knee joint extensor and flexor torques, DJ, SHD and 6MTH are illustrated as means ± standard deviations (M ± SD). *P* values and effect sizes (partial eta square (η^2^_p_)) are given for dominance*sex interaction effects

**Fig. 3:**
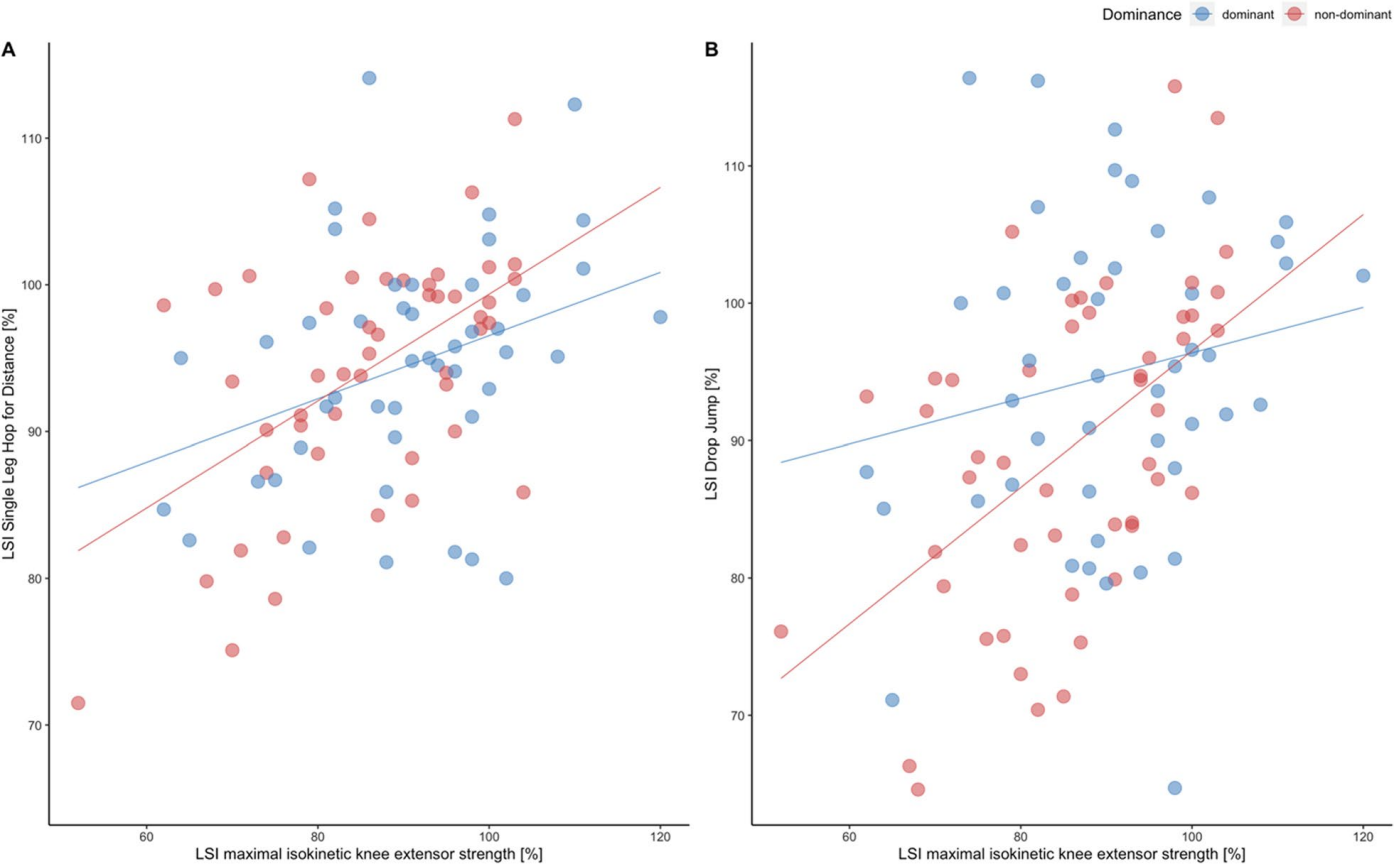
In panel** A**, a scatterplot with regression line of LSI maximal isokinetic knee extensor strength [%] and LSI DJ [%] is presented. In panel** B**, a scatterplot with regression line of LSI maximal isokinetic extensor strength [%] and LSI SHD [%] is illustrated. Red dots indicate non-dominant and blue dots the dominant leg. The shaded area represents 95% confidence interval

Simple linear regression analysis was performed to elucidate the predictive effects of knee extensor strength on LSI. The results for the whole cohort demonstrated that LSI maximal isokinetic knee extensor strength was a significant predictor of performance for the LSI SHD (adjusted R^2^ = 18.2%, *p* < 0.01, Fig. 3A) and LSI DJ (adjusted R^2^ = 14%, *p* < 0.01, Fig. 3B). For each percentage increase in LSI maximal isokinetic knee extensor strength, an increase of 0.4% in LSI DJ and an increase of 0.3% in SHD can be expected according to the respective regression equations.

## Discussion

This study permits major insights into the interrelationship of limb dominance, sex and the LSI of functional performance tasks at the time of return to sport in patients following primary arthroscopic reconstruction of the torn ACL. We found that nine months after surgical reconstruction (i) limb dominance significantly influences the LSI of the knee extensor strength with higher LSIs in patients who injured the dominant leg than for those who injured the non-dominant leg (Table 2). (ii) Sex revealed no significant effects (Table 3 and Fig. 2). Additionally, (iii) the LSI of maximal isokinetic knee extensor strength was a significant predictor of performance for the LSI for drop jump and single-leg hop for distance (Fig. 3).

Based on these findings, we suggest clinicians to take limb dominance into account when return to sport decisions following ACL reconstruction are made from LSI evaluation. Although a *successful* return to sport was not investigated within the current study, the present data indicate a potential overestimation of LSI, when the dominant leg was injured. This might falsely lead to an accelerated return to sport in clinical practice which needs to be further investigated in future studies. Frequently used cut-off scores of 90% [10–13, 15] should be differentiated whether the dominant or non-dominant limb is injured; if the dominant limb is injured the patient should reach at least an LSI of 95% as suggested earlier by Fitzgerald [44]. Vice versa, a surgical reconstruction of the non-dominant leg requires a more generous LSI of approximately 85% for cut-offs that may be reached within the nine months rehabilitation process with reference to the dominant leg.

Procedures and cut-off values different from that aforementioned can be misleading. Dealing with incorrect reference values may either significantly shorten or lengthen the rehabilitation process only biased by years of experience and practice; Limb dominance is an important aspect in soccer (stance leg vs. kicking leg), long or high jump (jump leg vs. stem leg), combat sports (judo fight position) and many other sports. Frequent training naturally differentiates the legs with the muscles encompassing the knee joint [45]. Thereby, forces and coordinative skills are often differently developed and specialized over years and are not related to maladaptive asymmetries.

It is noteworthy, that monoarticular knee extensor strength predicts functional multiarticular performance during ballistic DJs and SHD. This interrelationship has a primary importance for return to sport when high impact, energy-absorbing landings or ballistic movements are required. For practitioners, the regain of maximal quadriceps strength is therefore of major importance during rehabilitation as it forms the basis for complex multiarticular performance which is highly relevant in the majority of sport disciplines. This interrelationship and aforementioned practical framework in the rehabilitation process are in accordance with findings of Schmitt et al. who demonstrated that extensor strength deficits negatively affect function and performance at the time of return to sport following ACL reconstruction [46]. In terms of knee flexion strength, deficits following ACL reconstruction might be even more pronounced especially when hamstring autografts are used during surgery [47].

Regarding the effect of limb dominance on the LSI, the results of this study are supported by findings of McGrath et al.’s review [48] with a total of 264 patients and Boo et al. [49]. They also did not find any statistical effect of limb dominance for the SHD test. About the underlying causes can only be speculated: differences in complexity and coordinative difficulty could make the SHD and familiar tasks like the 6MTH less sensitive for the limb dominance. McGrath et al. [48] did not reveal a significant difference for extensor quadriceps strength, although there was a non-statistical trend towards a postoperatively higher strength when the dominant leg was injured. Contrary findings can be due to differences in statistical procedures: our study calculated the LSI to eliminate inter-subject confounders whereas McGrath et al. [48] analyzed raw data.

Even though the methodological approach in the current paper was carefully chosen based on previous evidence, further limiting aspects could not be ruled out. Despite the large sample size in the present study, our population included a higher number of males (ratio of 2:1) which led to small samples for the gender stratified subgroup analysis and therefore does not conclusively finalize the discussion of applying different benchmarks for the female population. With reference to our statistical model, no significant differences were observed between men and women (see also Additional file 1). However, this study is representing a sample which is common in orthopedic clinics and physiotherapies. Baseline measurements to detect adaptive or maladaptive asymmetries and comparison with post-operative outcomes are missing. Due to the retrospective character of the study, there was no documentation of the activity level of the included patients, which could have helped to further calculate potential confounding effect on hop test performance. Lastly, although the present study identified LSI maximal isokinetic knee extensor strength as a predictor of the LSI during DJ (adjusted R^2^ = 14%) and SHD (adjusted R^2^ = 18.2%), further studies need to investigate the clinical relevance of this relationship.

Despite being a widely used tool in return to sport assessment, the sole use of LSI without further additional measures might overestimate knee function [25] and might therefore be used in conjunction with further rigorous testing criteria.

## Conclusions

This cross-sectional study demonstrates the dependency of the LSI from the knee extensor strength from limb dominance. Thereby, higher LSIs of the knee extensor strength are associated with higher drop jump and single hop for distance performances. Considering limb dominance when interpreting LSI for return to sport is a therapeutical prerequisite and paramount to lower the injury risk for ACL reconstruction patients. Patients who injured their dominant limb should be rated more strictly with a higher LSI cut-off level than patients who injured the non-dominant limb. Limb dominance is only one of the factors influencing the LSI calculation before return to sport. Further studies are required to investigate other factors like primary sport, general fitness level, psychological aspects (motivation or anxiety) that may potentially affect LSI.

## Supplementary Information

**Additional file 1**. Detailed regression parameters for statistical models used within the present study. 

## Data Availability

The datasets used and/or analysed during the current study are available from the corresponding author on reasonable request.
