# Supplementary material for: How limb dominance influences limb symmetry in ACL patients: effects on functional performance
Source: BMC Sports Sci Med Rehabil. 2022 Dec 7;14:206. doi: 10.1186/s13102-022-00579-y (PMC9727863; doi:10.1186/s13102-022-00579-y)

**Additional File 1**

**Table 1** Regression parameters for LSI maximal isokinetic knee extension strength


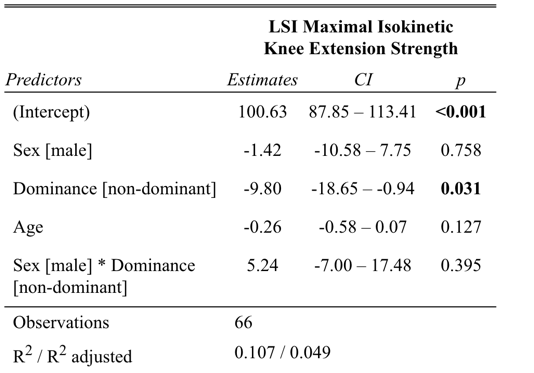


**Table 2** Regression parameters for LSI maximal isokinetic knee flexion strength


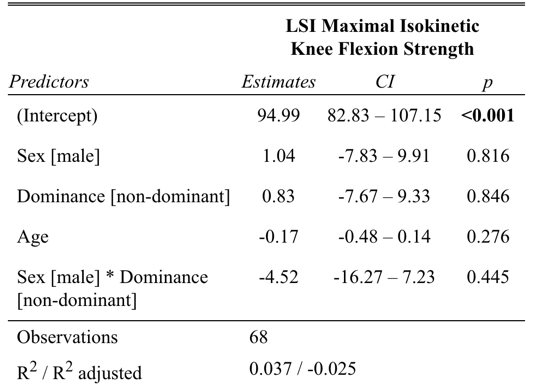


**Table 3** Regression parameters for LSI drop jump


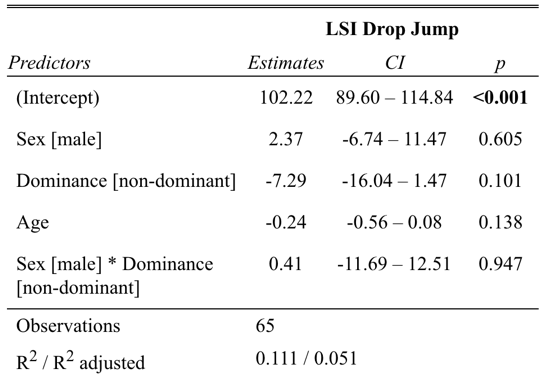


**Table 4** Regression parameters for LSI single leg hop for distance


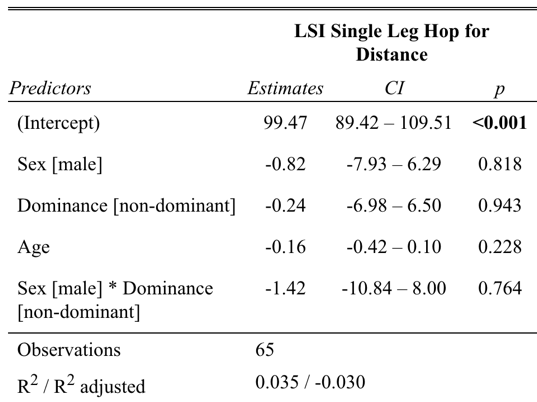


**Table 5** Regression parameters for LSI 6m timed hop


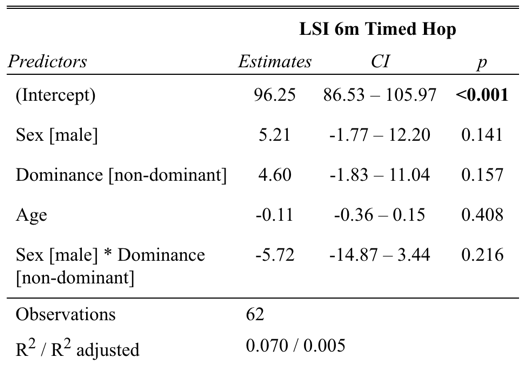

Supplement: Supplementary file 1 — Additional file 1. Detailed regression parameters for statistical models used within the present study. [file 13102_2022_579_MOESM1_ESM.docx]
